# Development of nomograms predictive of anastomotic leakage in patients before minimally invasive McKeown esophagectomy

**DOI:** 10.3389/fsurg.2022.1079821

**Published:** 2023-01-26

**Authors:** Jianqing Chen, Jinxin Xu, Jianbing He, Chao Hu, Chun Yan, Zhaohui Wu, Zhe Li, Hongbing Duan, Sunkui Ke

**Affiliations:** ^1^ Department of Thoracic Surgery, Zhongshan Hospital Xiamen University, Xiamen, China; ^2^ Department of Thoracic Surgery, Fuqing City Hospital, Fuqing, China

**Keywords:** esophagectomy, anastomotic leakage, predictive model, nomogram, aortic calcification

## Abstract

**Purpose:**

The present study aims to identify factors related to anastomotic leakage before esophagectomy and to construct a prediction model.

**Methods:**

A retrospective analysis of 285 patients who underwent minimally invasive esophagectomy (MIE). An absolute shrinkage and selection operator was applied to screen the variables, and predictive models were developed using binary logistic regression.

**Results:**

A total of 28 variables were collected in this study. LASSO regression analysis, combined with previous literature and clinical experience, finally screened out four variables, including aortic calcification, heart disease, BMI, and FEV1. A binary logistic regression was conducted on the four predictors, and a prediction model was established. The prediction model showed good discrimination and calibration, with a C-statistic of 0.67 (95% CI, 0.593–0.743), a calibration curve fitting a 45° slope, and a Brier score of 0.179. The DCA demonstrated that the prediction nomogram was clinically useful. In the internal validation, the C-statistic still reaches 0.66, and the calibration curve has a good effect.

**Conclusions:**

When patients have aortic calcification, heart disease, obesity, and a low FEV1, the risk of anastomotic leakage is higher, and relevant surgical techniques can be used to prevent it. Therefore, the clinical prediction model is a practical tool to guide surgeons in the primary prevention of anastomotic leakage.

## Introduction

Esophageal carcinoma is one of the most common malignant tumors in the upper gastrointestinal tract. In 2020, the total number of new cases and deaths from esophageal cancer worldwide were 604,100 and 544,076, respectively, with its morbidity and mortality rates ranking 7th and 6th among all malignant tumors (1).

Anastomotic leakage (AL) after esophagectomy is what patients must consider a frequent and severe postoperative complication, which leads to a prolonged length of hospital stay (2, 3), increased physical and psychological distress, and even a delay in postoperative adjuvant therapy, resulting in an increased risk of distant metastasis of the tumor (4). With the development of surgical techniques and perioperative patient management, the incidence of anastomotic leakage is lower than before. However, according to a recent analysis of 6,022 patients from the Esodata dataset, who underwent esophageal resections at 39 centers representing 19 countries between January 2015 and December 2018, the frequency of leaks remains high, with an incidence rate of 12.5% (5). Therefore, early clinical observation and identification of anastomotic leakage are very important.

The current medical model has been transformed from traditional experience medicine to evidence-based medicine and gradually developed into precision medicine. As data are easier to obtain and predictive analysis becomes more convenient, the value of clinical data has received unprecedented attention, and individualized medicine has been mentioned more and more by clinicians (6). The clinical prediction model, as a quantification tool for assessing risks and benefits, can help doctors and patients make decisions before the outcome is available. The nomogram is a simple tool for predicting complications in clinical practice (7). It graphically compares known factors and makes individualized risk prediction more concise and intuitive.

Although many studies exist on the risk of anastomotic leakage after esophagectomy worldwide, there is still a lack of specific methods to evaluate the risk of anastomotic leakage before surgery, which can guide the significance of preoperative and intraoperative intervention for patients. This study aimed to establish a practical clinical prediction model for evaluating anastomotic leakage preoperatively in esophageal carcinoma patients. Patients with a potentially high risk of anastomotic leakage were screened according to their general physiological conditions and preoperative examinations, and individualized clinical intervention and surgical plan adjustment were given to reduce the incidence of postoperative anastomotic leakage.

## Materials and methods

### Patients

A retrospective analysis was carried out on 557 patients who were diagnosed with esophageal cancer by pathology or cytology and treated for radical esophagectomy between January 2015 and January 2020. The inclusion criteria were as follows: gastroscopy was performed preoperatively, patients were pathologically confirmed to have esophageal cancer, a minimally invasive McKeown esophagectomy with stapled anastomoses, including a three-field lymphadenectomy, was performed, and resection was performed with negative resection margins (pR0). Patients who had recurrent or metastatic cancer, palliative resection due to the discovery of T4b or M1 disease during surgery, an organ reconstruction other than gastric tube reconstruction, a route reconstruction other than posterior mediastinal route, and incomplete clinical data were excluded. A total of 285 patients were included in this study, and 272 patients were excluded, including 13 cases with data deletion, 45 cases with Ivor-Lewis or Sweet surgery, 5 cases with colon reconstruction or jejunal reconstruction, 99 cases with manual anastomosis, 88 cases with two-field lymphadenectomy, and 22 cases with the retrosternal route. The study design was approved by the institutional review board and ethics committee of Zhongshan Hospital at Xiamen University. Patient consent for inclusion was waived owing to the use of identified retrospective data.

### Surgical procedures

Our standard procedures consisted of a three-field surgery (the modified McKeown procedure, with laparoscopy and right video-assisted thoracoscopic surgery) and reconstruction with a gastric tube through a posterior mediastinal route. An end-to-side esophagogastric anastomosis was performed in the neck using a circular stapled anastomotic technique. Lymph node dissection was based on a total three-field lymphadenectomy. The extent of the three-field LN dissection, including all nodes and periesophageal tissues below the level of the carina to the celiac trifurcation and all superior mediastinal nodes along the recurrent laryngeal nerve to the lower poles of the thyroid and lymph nodes in the supraclavicular fossa.

### Potential predictor variables

The selection of candidate predictors was based on the reference literature and relevant clinical experience reported in Table 1. Intra-operative data and the pathological result were not included in the candidate predictors, although they were reported to be significant risk factors. In the clinic, preoperative evaluation for the incidence of anastomotic leakage is recommended, along with further interventional measures, including the selection of an appropriate surgical strategy and the extent of lymph node cleaning. Here, therefore, our patients included were given McKeown esophagectomy under thoracoscopy and laparoscopy plus a three-field lymph node dissection by two experienced thoracic surgeons, hoping to balance the effect of intra-operative variables on the incidence of anastomotic leakage. Because pathological reports can be obtained 5–7 days after surgery, the peak period of anastomotic leakage, they cannot be used for early leakage prediction. Given the condition, preoperative chest and abdominal CT imaging and gastroscopic puncture biopsy pathology are considered alternatives.

**Table 1:** Demographics and clinical characteristics Among 285 patients with minimally invasive McKeown esophagectomy.

Characteristic	AL	Non-AL
Total number	77	208
Age (years)	61.0 ± 8.2	59.9 ± 8.2
**Sex**		
Female	12 (15.6%)	37 (17.8%)
Male	65 (84.4%)	171 (82.2%)
BMI (kg/m^2^)	21.8 ± 3.1	21.4 ± 2.9
Smoking	48 (62.3%)	139 (66.8%)
Hypertension	24 (31.2%)	46 (22.1%)
Diabetes	6 (7.8%)	16 (7.7%)
Cardiac disease	6 (7.8%)	5 (2.4%)
Aortic calcification	33 (42.9%)	35 (16.8%)
nCRT	14 (18.2%)	49 (23.6%)
**Pathology**		
Squamous	74 (96.1%)	203 (97.6%)
Adenocarcinoma	1 (1.3%)	2 (1.0%)
Other	2 (2.6%)	3 (1.4%)
**Location**		
Upper	9 (11.7%)	38 (18.3%)
Middle	57 (74.0%)	128 (61.5%)
Lower	11 (14.3%)	42 (20.2%)
Long diameters (cm)	4.762 ± 3.546	4.58 ± 2.43
Total protein (g/L)	71.2 ± 5.3	70.5 ± 6.3
Albumin (g/L)	41.8 ± 3.5	41.8 ± 4.8
ALT (U/L)	18.3 ± 14.4	17.2 ± 11.1
AST (U/L)	20.7 ± 9.4	20.1 ± 9.1
Urea (mmol/L)	5.2 ± 1.8	5.4 ± 1.5
CREA (µmol/L)	76.3 ± 16.0	75.9 ± 15.6
Glu (mmol/L)	5.7 ± 1.8	5.5 ± 1.2
PLT (10^9^/L)	248.4 ± 83.8	247.2 ± 72.5
Hb (g/L)	133.9 ± 14.8	134.1 ± 15.9
HCT (%)	39.8 ± 4.4	39.9 ± 4.7
FEV1 (L)	2.7 ± 0.6	2.8 ± 0.7
FEV1% Pred	100.6 ± 18.5	101.4 ± 17.2
FVC (L)	3.5 ± 0.7	3.6 ± 0.8
FVC1% Pred	104.2 ± 14.7	105.5 ± 15.5
FEV1/FVC (%)	76.6 ± 9.9	76.9 ± 8.8
**Pulmonary function**		
Normal	53 (68.8%)	158 (76.0%)
Mild dysfunction	19 (24.7%)	40 (19.2%)
Moderate dysfunction	4 (5.2%)	7 (3.4%)
Severe dysfunction	1 (1.3%)	3 (1.4%)

AL, anastomotic leakage; BMI, body mass index (kg/m^2^); nCRT, Neoadjuvant chemoradiotherapy; FEV1, forced expiratory volume in the first second; FVC, forced vital capacity.

### Definitions of anastomotic leakage

We defined anastomotic leakage as a full-thickness GI defect involving esophageal anastomosis, a staple line, or both, irrespective of presentation and method of identification (8). Anastomotic leakage, if early, can happen at the end or within 3 days of operation, mainly attributed to inappropriate anastomotic techniques or operating methods. If late, anastomotic leakage may develop 2 weeks or even 1 month post operation, commonly around 1 week post operation. It is established that anastomotic leakage has three levels: mild, medium, and severe. For mild cases, no particular clinical manifestations are presented, and they are often diagnosed during an examination, with no need for medical treatment and delayed oral feeding discontinuation as a curable option. For medium cases, symptoms of sepsis can be clearly seen in gastroscopy, radiography, and a CT image, and clinical interventions are required, including anti-infection therapy, bedside incision opening and gauze filling, drainage, stent implantation, etc. While for severe cases, clinical symptoms present to be critical, requiring surgical treatment. In the present study, all patients received cervical anastomosis. In these cases, the neck skin manifests red and swollen, tenderness, subcutaneous emphysema, putrid pus when pressed, saliva or gastric juice-like substance seen in the neck drainage tube, or even symptoms like increased body temperature and heart rate, cervical anastomotic leakage is then suspected. Esophagus-chest enhanced CT can be implemented to identify the anastomotic leakage. If necessary, open the incision on the left neck to observe and conduct debridement for drainage. In circumstances where patients develop anastomotic leakage in routine DR 7 days after the operation with no local or systemic inflammatory responses, symptomatic treatment like fasting is given temporarily.

### Statistical analysis

Continuous variables were presented with a mean and standard deviation. Frequencies and percentages were presented using categorical variables. Statistical analysis was conducted using the R software (Rx64 4.0.12). The last absolute shrinkage and selection operator is a scenario favored in variable screening by the statistician (9). It was able to find an optimal equilibrium point between accuracy estimation for models and the absolute values of the coefficients. This algorithm regulated the penalty coefficient so that errors could be minimized to achieve the screenings purposes and avoid the problem of overfitting. In addition to screening variables by statistical methods, artificial addition or deletion of variables is allowed after approval by clinical experts. Then, risk factors selected from the Lasso analysis were assessed using a binary logistic regression modeling technique; Besides, a nomogram that can visualize the prognostic strength of different risk factors in a single figure was established. The concordance index (c-index) and calibration curve were used to determine its predictive accuracy and discriminatory capacity. The Brier score was used for overall performance and captures aspects of both calibration and discrimination. Decision curve analysis was used to assess the clinical impact of the prediction model by quantifying the net benefits at different threshold probabilities. Last, the internal validation of the nomogram was conducted by bootstraps with 100 resamples.

## Results

### Patient characteristics

During the study period, 557 consecutive patients who had a malignant tumor of the esophagus based on preoperative imaging and bioptic-based histopathology underwent esophagectomy. Of these, 285 patients [236 males and 49 females; mean age 60 ± 8.2 years (range 36–87 years)] who met the inclusion were enrolled. The incidence of anastomotic leakage after esophagectomy in this study was 27%, which was comparable and slightly higher than previous reports (rate of anastomotic leakage between 8% and 35%) (10, 11). Patients were divided into an AL group and a non-AL group. The demographic and clinical characteristics of the patients are summarized in Table 1.

In order to exclude the influence of intraoperative factors, the results of non-parametric test analysis and chi-square analysis showed no significant difference between the groups with and without anastomotic leakage after esophagectomy in terms of the surgeon, operative time, and intraoperative estimated blood loss (Table 2).

**Table 2:** Intraoperative parameters of 285 patients with minimally invasive McKeown esophagectomy.

Variable	AL	Non-AL	*P*
Surgeon A	125 (60.10%)	48 (62.34%)	0.731
Surgeon B	83 (39.90%)	29 (37.66%)	
Surgery time (min)	381.2 ± 110.0	360.0 ± 101.9	0.107
Blood (ml)	170.8 ± 133.5	174.7 ± 142.1	0.689

AL, anastomotic leakage.

### Development and evaluation of the predictive model

**Figure 1:**
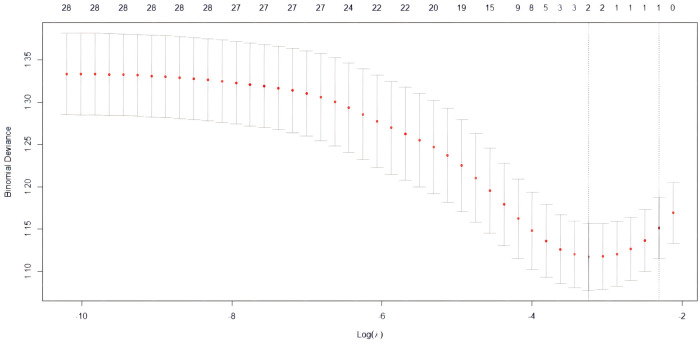
The LASSO regularization parameter lambda was selected by 10-fold cross-validation using the cv.glmnet function, and the optimal Lambda value was identified by the minimum cross-validated criterion and the minimum criterion within one standard error.

**Figure 2:**
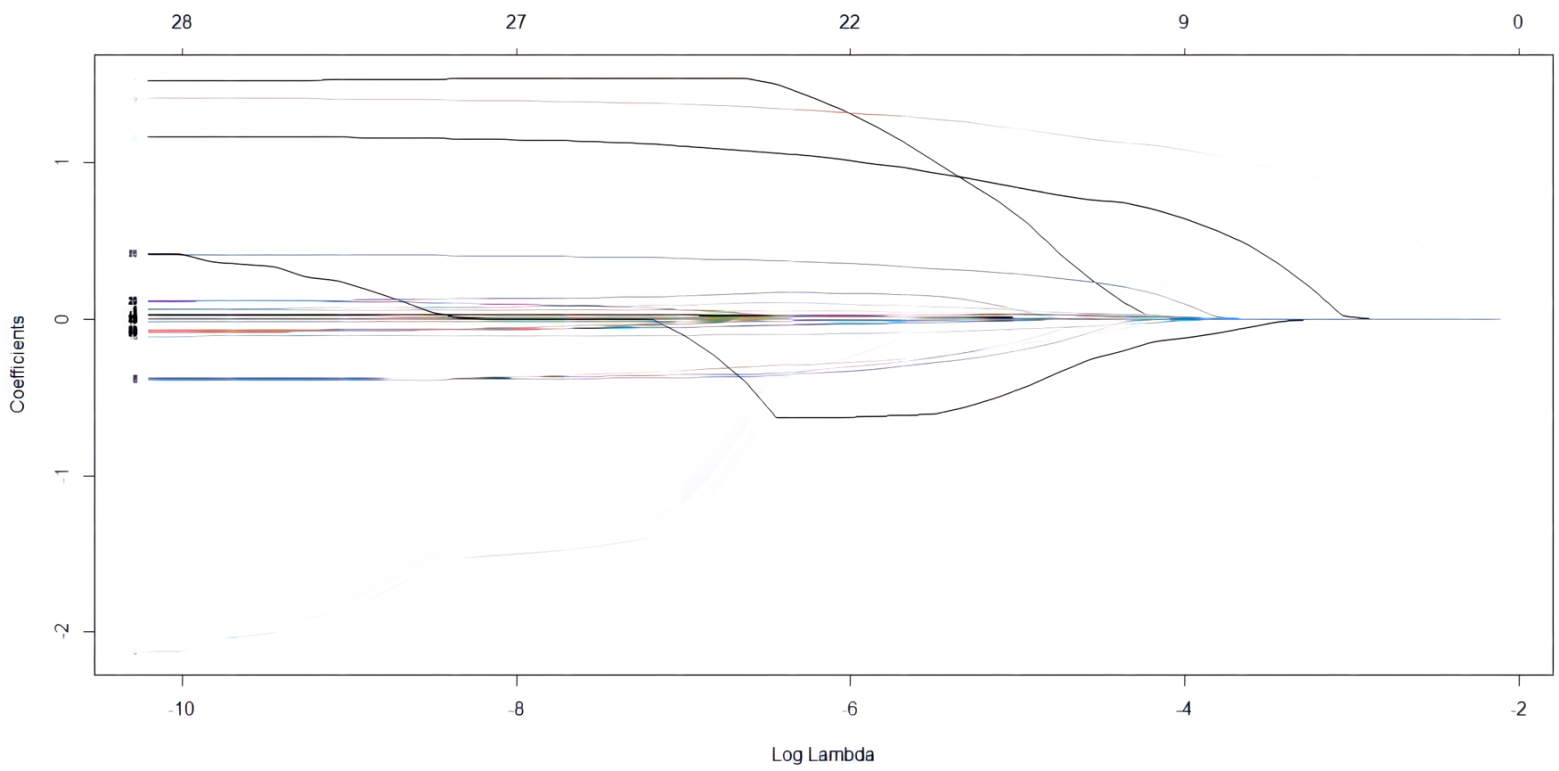
LASSO coefficient profiles of 28 predictive risk factors according to log(Lambda) sequence.

**Figure 3:**
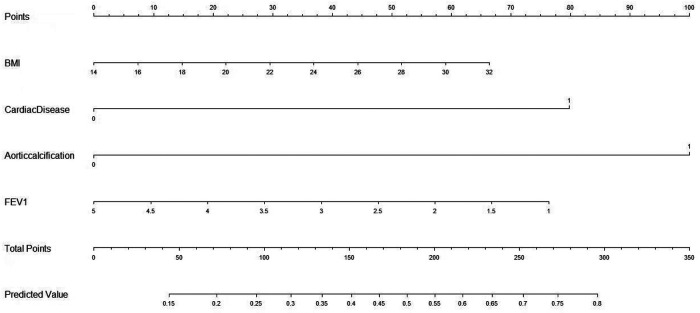
The developed AL risk nomogram with minimally invasive McKeown esophagectomy.

Lasso regression was performed on 285 cases with 28 clinical characteristics and demographic information using R software. Then, two predictors with non-zero coefficients were screened (Figures 1, 2), which were aortic sclerosis and heart disease, and aortic sclerosis (*P* < 0.05) was statistically significant. Based on the literature and previous clinical experience, we additionally added body mass index (kg/m^2^) (BMI) and forced expiratory volume in the first second (FEV1) as predictors to the analyses. Anastomotic leakage was used as the dependent variable, and aortic sclerosis, heart disease, BMI, and FEV1 were included as independent variables in a binary logistic regression model. We used the following formulas for the logistic model to calculate the probability: probability = 1/(1 + *e*^−Y^), *e* = base of the natural logarithm, *Y* = −1.73791 + (0.04572 × BMI) + (0.98899 × heart disease) +(1.23872 × aortic sclerosis) − (0.23648 × FEV1). Then, the nomogram to predict anastomotic leakage was plotted using R software for visualization purposes (Figure 3).

**Figure 4:**
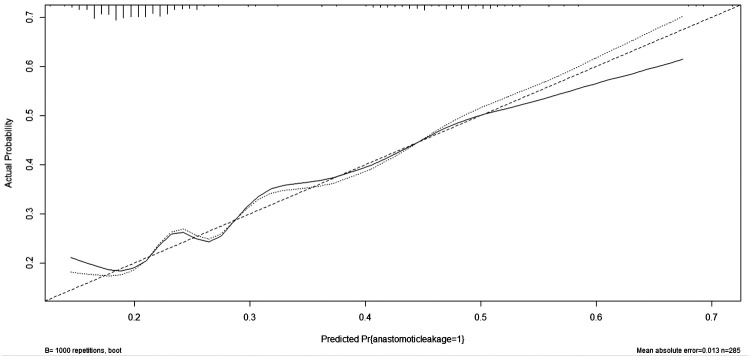
The accuracy of the model for identifying patients with AL was determined using AUC analysis.

**Figure 5:**
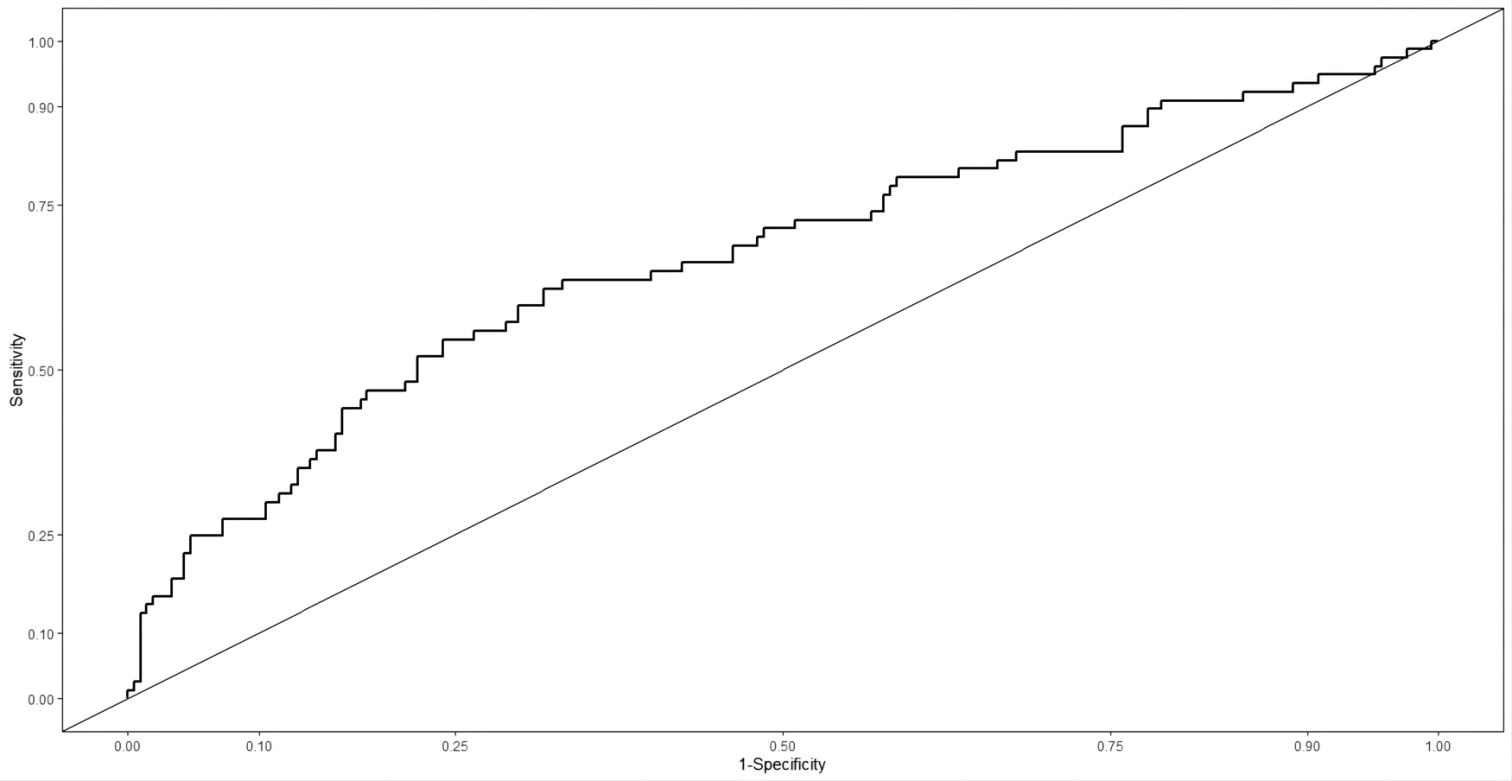
Calibration plots of the nomogram. The solid line represents the bias-corrected performance of the nomogram, where a closer fit to the diagonal dotted line represents a better prediction.

**Figure 6:**
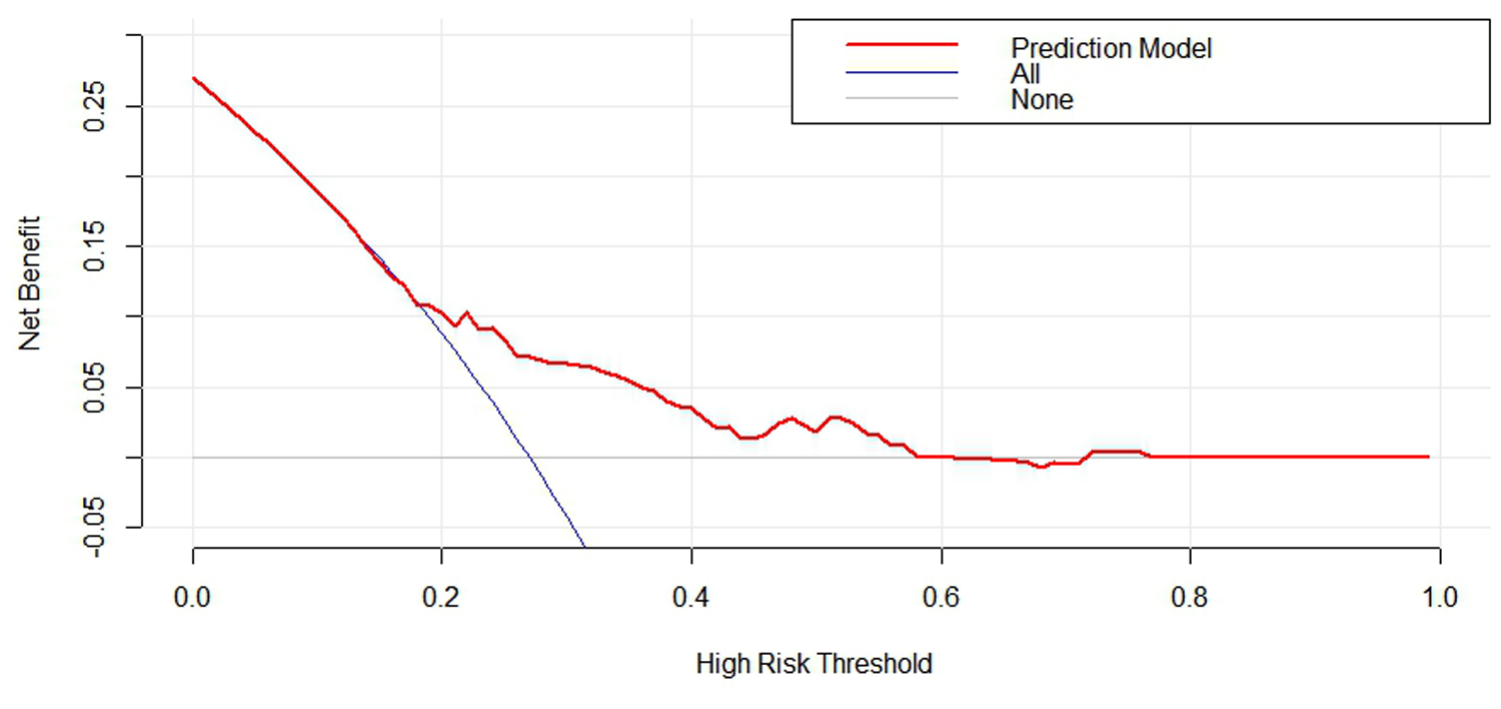
Decision curve for nomogram to predict the risk of anastomotic leakage before esophagectomy. The blue line assume all patients have AL. The gray line assume no patients have AL. The red curve represent clinical benefits of patients at different risk levels of AL.

The predictive accuracy of the model was assessed using (1) C-index for discrimination, which measures how well the model discriminates between patients with and without AL. The predictive nomogram achieved a C-index of 0.67 (95% confidence interval [CI], 0.593–0.743) as outcome events are a dichotomous categorical variable, the same as in the area underthe ROC curve (AUC), And the receiver operating characteristic curve is displayed in Figure 4; (2) A calibration curve was based on the actual incidence and predicted incidence. The dotted line represents *y* = *x* which means that the predicted and measured rates are exactly the same. The calibration curve of the nomogram to predict AL risk before oesophageal surgery demonstrated good agreement in this cohort (Figure 5), and (3) Brier score for overall performance, which ranges from 0 to 1, with a value closer to 0 indicating better predictive ability, and our model score of 0.179. The decision curve showed that if the threshold probability of a doctor is between 18% and 60%, using the nomogram to predict AL adds more benefit (Figure 6). Bootstrapping with 100 repetitions was used for model validation, and the bias-corrected measure of accuracy was c-index of 0.66. Together, the values we obtain for these measures indicate reasonably good predictive accuracy and are clinically useful.

### Use of the nomogram

The steps for using the nomogram are determining the patient's value for each predictive variable, adding the points from each predictor according to the top point reference line, and locating the sum of the points on the total points axis, which corresponds to the patient's likelihood of having AL.

The applicability of the nomogram can be illustrated through a clinical example: If a patient with a BMI of 22 kg/m^2^, FEV1 of 4.5 L, aortic calcification, no previous history of heart disease, according to the nomogram, scores of each predictor were calculated to be 30, 77, 100, and 0, the total points would be 207, and the risk of AL would be 57%. The expected likelihood of AL for individual patients can be used for preoperative counseling and treatment planning.

## Discussion

Esophageal cancer is a common malignant tumor of the digestive tract that occurs in the esophageal epithelium. Surgery is the main treatment option for esophageal cancer, and anastomotic leakage is one of the most serious complications after esophageal cancer resection. Therefore, patients with esophageal cancer may benefit from the early prevention and detection of AL.

In recent years, with the popularization of statistical methods for clinical prediction models, more and more surgeons have applied them to the prediction of postoperative complications. Huang et al. and Sun et al. analyzed various indicators of the perioperative period, including the general state of patients, anastomosis site and method, postoperative blood inflammation index, and complications, to establish a risk prediction model for anastomotic leakage after esophagectomy, respectively (12, 13). In this case, the researcher systematically identified and rated their performance, a presentation we have not found in previous literature. This model provided a reference for doctors to diagnose anastomotic leakage in patients following esophagectomy. Unfortunately, due to the inclusion of intraoperative and postoperative indexes, these models simply apply to predict the incidence of anastomotic leakage after surgery and cannot advise for the prevention of anastomotic leakage preoperatively targeted at surgical methods, sites of anastomosis, and the extent of the lymph node dissection.

Previous studies have mainly focused on surgical factors and postoperative data, which is quite different from ours. We developed a novel tool to predict the healing ability of the anastomosis in the stomach conduit before esophageal surgery based on 5 years of data from indigenous Chinese patients. The main advantage of the current study is that our nomogram mainly applies to preoperative assessment, offers individualized surgical strategies, and achieves the goal of primary prevention. Four of the 28 clinical parameters were screened, and the weighting of each parameter was significant in the nomogram, which could reflect the significant influence of these factors on the predicted value.

Atherosclerosis, now the recognized trigger for tissue ischemia, is inferred to have an impact on the anastomotic blood supply of the gastric tube (14). A previous study used the vascular calcification of arteries as an indicator for atherosclerosis to predict the risk of cardiovascular events (15). Inspired by this, van Rossum et al. proposed a semi-quantitative scoring system, which is practicable in evaluating the vascular calcification of gastric feeding arteries, based on preoperative chest and abdominal CT images of patients suffering esophageal carcinoma (16). The research displayed that the vascular calcification of gastric feeding arteries in preoperative routine chest and abdominal CT images was associated with the risk of cervical anastomotic leakage post radical treatment for esophageal carcinoma, and the calcification of aorta and common hepatic artery were identified as independent risk factors (*P* < 0.05). Such a finding is supported by anatomy and pathophysiology and is evidenced by the right gastroepiploic artery, which is derived from the branch of the common hepatic artery and serves as a supplier of gastric tube and anastomotic blood. By now, however, it is still a mystery whether the association of anastomotic leakage with arterial vascular calcification is only present in the limited blood flow of the gastric tube induced by local vascular disease or is also applicable in systemic vascular disease. In order to clarify the underlying relationship, Borggreve et al. conducted an analysis of the clinical information of 406 cases and then scored the arterial calcification from 10 positions throughout the body by CT imaging (17). As analyzed, the calcification of coronary arteries and aorta-arch superior thyroid arteries (brachiocephalic trunk, right common carotid artery, and right subclavian artery) were independent risk factors for anastomotic leakage. Such calcification may be a the predisposing factor or the outcome of diffuse arterial diseases. Hence, it can help identify patients who have a risk of anastomotic leakage. As such, there was a retrospective study by Goense et al. devoted to 167 esophageal carcinoma cases after the operation, indicating that the existence and severe degree of thoracic aorta calcification were associated with the risk of anastomotic leakage post-esophagectomy in an independent manner (18). This is in agreement with our findings. Here, only the right gastroepiploic arteries were retained following the McKeown esophagectomy, making the bottom of the gastric tube suffer from a relatively deficient blood supply, while vascular calcification might further aggravate the condition. Notably, vascular disease is tightly associated with multiple systemic and chronic lesions, such as diabetes, peripheral vascular disease, and renal insufficiency, which might be involved in the cure of cervical anastomosis via various pathways.

Heart-relevant diseases are defined as previous coronary atherosclerotic heart disease, continuous arrhythmia, a history of organic heart lesions, and abnormal diseases reflected in an improved electrocardiogram and echocardiography at admission. Additionally, the unstable hemodynamics during and post operation induced by heart-related diseases is as well a risk factor for anastomotic leakage. A meta-analysis by Schizas et al. revealed patients with atrial fibrillation had a significantly increased risk of anastomotic leakage relative to patients without atrial fibrillation (OR = 2.65, 95% CI, 1.53–4.59) (19). This might be attributed to the unstable hemodynamics caused by atrial fibrillation, leading to decreased anastomotic tissue blood, ultimately resulting in gastric tube ischemia and anastomotic leakage. The development of postoperative atrial fibrillation is partially due to the close range between the esophagus and left atrium in anatomy, and the free esophagus around the pericardium can increase the risk of left atrium associated complications during the operation. While coronary atherosclerosis and organic heart disease are recognized factors leading to atrial fibrillation.

Sufficient tissue oxygen delivery is another prerequisite for a smooth anastomotic cure (20). Compelling evidence by Gao et al. on 129 esophageal carcinoma cases who undertook minimally invasive McKeown operation indicated that the preoperative FEV1 <2.18 L and the lowest intra-operative ABG PaCO2 >45.5 mmHg were risk factors of anastomotic leakage post operation (21). The research here demonstrated that the lower preoperative FEV1 reflected the higher incidence of postoperative anastomotic leakage. Studies in the past have noted that factors such as COPD, near-term smoking, and pneumonia, which are responsible for decreased pulmonary function, are also risk factors for cervical anastomotic leakage (22–24). This might be attributed to the low anastomotic tissue oxygenation associated with poor pulmonary function during and post operation. In view of the above, we should pay more attention to the association between pulmonary function and postoperative complications. In addition, active pulmonary function exercise, absolute smoking cessation for at least 2 weeks, atomization, reducing sputum, and other clinical interventional measures can help reduce the incidence of pneumonia and anastomotic leakage post operation.

Body mass index (BMI), calculated as weight in kilograms divided by height in square meters (kg/m^2^), is a confirmed risk factor for anastomotic leakage. A meta-analysis by Mengardo et al. reports a higher incidence of AL in obese patients than in non-obese patients (25). Diabetes, dyspnea, and cardiac disease appeared significantly more prevalent among obese patients and increased in parallel with the extent of BMI. Notably, a BMI lower than 18.5 kg/m^2^ and weight loss of 5% or more during the 3 months before surgery are strong indicators of malnutrition, which are reported to be associated with an increased risk of anastomotic leakage after esophagectomy. Therefore, underweight patients may benefit from preoperative nutritional assessments and nutritional supplementation due to their higher risk of malnutrition and cachexia. Overall, obese and underweight patients should receive extra attention for the early detection and prompt treatment of anastomotic leakage. In the present study, the nutritional risk score (NRS-2002) should be performed to screen for undernourished patients who would benefit from enhanced nutritional support preoperatively.

Studies have shown that neoadjuvant therapy affects the overall nutritional status of patients, their incredible immune function (26), increases the risk of postoperative infection, and ultimately has a negative impact on anastomotic healing. However, in the study, esophagectomy was performed in 285 patients, and 22.2% of patients with preoperative neoadjuvant therapy had AL. But 28.4% of patients without preoperative neoadjuvant therapy had AL (*P* = 0.33), and the results showed that neoadjuvant therapy was not associated with AL. However, some studies showed contrasting results (27, 28). Preoperative neoadjuvant therapy showed a correlation with AL. These studies suggest that patients receiving neoadjuvant therapy have more postoperative complications and a greater impact on cardiopulmonary function (29), which, in turn, reduces tissue perfusion and increases the risk of poor anastomotic healing. The differences in indications for neoadjuvant chemotherapy, choice of chemotherapy agents, and methods of operation may cause differences in the results.

With regard to these suggested causes, different attempts to optimize the conditions of anastomosis have been reported. A novel risk score for the prediction of anastomotic leakage may improve preoperative optimization, intraoperative strategy, and postoperative management. Prior to surgery, this nomogram offers a useful tool for clinicians to assess the risk of AL in individuals. Surgeons can then inform the patient and the referring physician of the predictive risk. Additionally, this new model plays instructive roles for surgical protocols in esophageal cancer patients. In the case of AL, some technical tips can be used prophylactically for high-risk patients. The use of pedicled omental transposition is a common surgery for the prevention of anastomotic leaks in carcinoma of the esophagus. The ability of the omentum to localize potentially dangerous inflammatory processes and induce neovascularization in the underlying tissues makes it a unique structure for preventing esophagogastric anastomotic leaks. In a previous study, Bhat et al. proved that the use of mobilized omentum wrapped around the anastomosis markedly decreased the incidence of anastomotic leakage, which has been evaluated in a prospective controlled trial (30). However, more accurate measurements and cutting is required before transposition. Surgery was difficult because some minor deviations may negatively affect the quality of pedicled omental. Song et al. adopted a novel approach using polymeric materials, requiring only proper tailoring during the operation. They reported excellent results, with a 2.4% incidence rate of anastomotic leaks and a 9.2% incidence rate using pedicled omental. Such results are attributed to the omental’s inability to form the tight separation layer after fat liquefaction, which leads to an increased risk of anastomotic infection and leakage, while polymeric materials serve as an effective isolation layer to prevent anastomotic bleeding and inflammatory exudation.

There are some limitations that need to be mentioned in this study. First of all, this study was a retrospective study conducted in a single high-volume institution, so selection bias cannot be completely excluded, and external validation is required by more large-scale multicenter studies. Second, there are still some data that have not been collected in this study, so we cannot exclude some potential confounders that are not included in the analysis. Therefore, clinical predictive models needed an appropriate number of influential factors that were easy to collect and use to predict outcome variables. But with the development of science and technology, we will continue to explore big data, machine learning, artificial intelligence, and other technologies to apply them in clinical practice to achieve precision medicine.

## Conclusion

In summary, when patients have aortic calcification, heart disease, obesity, and low FEV1, the risk of anastomotic leakage is higher. Identifying patients at risk of anastomotic leakage and providing relevant surgical techniques may help prevent postoperative complications. Therefore, the clinical prediction model in this study is a practical tool to guide surgeons in the primary prevention of anastomotic leakage in clinical practice.

## Data Availability

The original contributions presented in the study are included in the article/Supplementary Material, further inquiries can be directed to the corresponding author.
